# CD163+ M2c-like macrophages predominate in renal biopsies from patients with lupus nephritis

**DOI:** 10.1186/s13075-016-0989-y

**Published:** 2016-04-18

**Authors:** Gregor Olmes, Maike Büttner-Herold, Fulvia Ferrazzi, Luitpold Distel, Kerstin Amann, Christoph Daniel

**Affiliations:** Department of Nephropathology, Institute of Pathology, Friedrich-Alexander-University Erlangen-Nürnberg (FAU), Krankenhausstr. 8-10, 91054 Erlangen, Germany; Institute of Human Genetics, FAU Erlangen-Nürnberg, 91054 Erlangen, Germany; Department of Radiation Oncology, FAU Erlangen-Nürnberg, 91054 Erlangen, Germany

**Keywords:** Lupus nephritis, Macrophages, T-reg-cells, Macrophage subtypes

## Abstract

**Background:**

The role of macrophages in the pathogenesis of lupus nephritis, in particular their differentiation to a certain subtype (e.g., M1- or M2-like) modulating the inflammatory reaction, is unknown. Here we investigated whether the differentiation in M1- or M2-like macrophages depends on the stage of lupus nephritis and whether this correlates with clinical parameters.

**Method:**

Using immunohistochemical analysis we analyzed renal biopsies from 68 patients with lupus nephritis (ISN/RPS classes II–V) for infiltration with M1-like (iNOS+/CD68+), M2a-like (CD206+/CD68+), M2c-like macrophages (CD163+/CD68+), and FoxP3+ regulatory T-cells. In addition, clinical parameters at the time of renal biopsy, i.e., blood pressure, proteinuria and serum urea were correlated with the macrophage infiltration using the Spearman test.

**Results:**

The mean number of CD68+ macrophages was related to the diagnosed ISN/RPS class, showing the highest macrophage infiltration in biopsies with diffuse class IV and the lowest number in ISN/RPS class V. In all ISN/RPS classes we detected more M2c-like CD163+/CD68+ than M2a-like CD206+/CD68+ cells, while M1-macrophages played only a minor role. Cluster analysis using macrophage subtype numbers in different renal compartments revealed three main clusters showing cluster 1 dominated by class V. Clusters 2 and 3 were dominated by lupus class IV indicating that this class can be further differentiated by its macrophage population. The number of tubulointerstitial FoxP3+ cells correlated with all investigated macrophage subtypes showing the strongest association to numbers of M2a-like macrophages. Kidney function, as assessed by serum creatinine and serum urea, correlated positively with the number of total CD68+, M2a-like and M2c-like macrophages in the tubulointerstitium. In addition, total CD68+ and M2c-like macrophage numbers highly correlated with Austin activity score. Interestingly, in hypertensive lupus patients only the number of M2a-like macrophages was significantly increased compared to biopsies from normotensive lupus patients.

**Conclusion:**

M2-like macrophages are the dominant subpopulation in human lupus nephritis and particularly, M2a subpopulations were associated with disease progression, but their role in disease progression remains unclear.

## Background

Lupus nephritis is the most relevant manifestation of a heterogeneous group of autoimmune disorders appearing in up to 60 % of patients with systemic lupus erythematosus (SLE), with varying degrees of renal involvement [1]. Currently, according to the International Society of Nephrology/Renal Pathology Society (ISN/RPS) system lupus nephritis is classified into six different classes with subclasses, using characteristic histological findings, i.e., class I, minimal mesangial lupus nephritis; class II, mesangial proliferative lupus nephritis; class III, focal lupus nephritis; class IV: diffuse segmental or global lupus nephritis; class V, membranous lupus nephritis, and class VI, advanced sclerosing lupus nephritis [2]. Major mechanisms initiating the inflammatory response in lupus nephritis are the production of autoantibodies and complement activation, but cellular immune mechanisms mediated through infiltrating mononuclear cells including macrophages have an important role in amplification and progression of renal injury in SLE [3]. Much work has been done to elucidate the pathophysiology of and optimal therapy for SLE, focused on the role of autoimmune B cells, T cells, and plasma cells. Recently, it has become evident, that macrophages are also important players in the pathogenesis of lupus nephritis. Fc-receptor-bearing myeloid cells, including macrophages, are responsible for triggering murine lupus nephritis [4]. Macrophages are involved in the clearance of apoptotic cells, which is known to be inefficient in SLE patients, resulting in the presence of, e.g., nuclear antigens that elicit autoantibody production [5]. In addition, macrophages activate other immune cells in inflammatory diseases by secretion of different cytokines [6]. Macrophages comprise a heterogeneous population of cells with different phenotypes and functions [7]. In the past macrophages were identified as promoting the progression of kidney disease. Macrophage-depleting strategies or interventions that interact with the macrophage chemokine system have been successful in ameliorating different inflammatory kidney diseases [8–11], including models for lupus nephritis [12–16]. Similar to T cells, macrophages can polarize to a specific subtype with completely opposed functions ranging from pro-inflammatory to anti-inflammatory or pro-fibrotic actions. Macrophage subtypes can be characterized by investigation of marker profiles and by measuring the cytokine expression. The latter method is more precise but is restricted to in vitro assays. A first classification of macrophage subtypes has been suggested by Mantovani et al. [17]. In this simplified classification, macrophages can be divided into two major classes: the classical activated M1-like macrophages, representing a pro-inflammatory type expressing the marker inducible nitric oxide synthase (iNOS) [18] and the alternatively activated M2-like-macrophages, which can be subdivided into M2a-like macrophages that are involved in repair and progression of fibrosis [19], M2b-like macrophages that are inducible by immune complexes and lipopolysaccharide (LPS) regulating immune response [20], and M2c-like macrophages exerting anti-inflammatory and pro-fibrotic actions [21].

Despite the investigation of macrophage polarization in different lupus mouse models [13, 22–24] and extensive discussion of the possible contribution of macrophage subtype to disease pathogenesis in human lupus nephritis [25], there are no data on the frequency of different macrophage subpopulations in human lupus nephritis. Therefore, we investigated renal biopsies from a cohort of 68 patients with SLE (ISN/RPN classes II–V) for macrophage subpopulations using immunohistochemical and cluster analysis.

In addition, infiltration of macrophage subtypes was correlated with renal morphological changes and clinical parameters to elucidate a potential impact of macrophage frequency and polarization on disease progression. To investigate the immune modulatory function of macrophages in lupus nephritis we also performed immunohistochemical analysis of forkhead-box-protein P3 (FoxP3)-positive T cells in renal biopsies.

## Methods

### Renal tissue specimens

In our study we analyzed macrophage polarization in 68 cases of lupus nephritis that were allocated to SLE classes II–IV according to the ISN-RPS [2]. All diagnoses were made by expert nephropathologists. An overview of the composition and clinical data for the patients with lupus nephritis is provided in Table 1.

**Table 1:** Characteristics of patients with systemic lupus erythematosus

	ISN/RPS class				
	II	III	IV	V	Sum/mean
Number of patients	8	13	26	21	68
Age, years	32.6 ± 18.7	32.3 ± 11.9	31.4 ± 14.6	36.7 ± 16.3	33.3 ± 15.0
Male	1	1	4	5	11
Female	7	12	22	16	57
Ratio male/female	0.14	0.08	0.17	0.31	0.19
Hypertensive patients, %	25	31	27	29	28
Diabetic patients, %	13	0	0	10	6
GSI	0.9 ± 0.7*	1.2 ± 0.7*	2.1 ± 0.5	1.1 ± 0.9*	1.3 ± 0.7
TSI	0.8 ± 0.6	1.4 ± 0.7	1.3 ± 0.4	1.0 ± 0.7	1.1 ± 0.6
Crescents, yes/no	0/8*	9/4	20/6	0/21*	29/38
Proteinuria, g/day	2.3 ± 3.2	3.0 ± 3.3	3.3 ± 2.4	4.8 ± 1.8	3.5 ± 2.7
Serum creatinine, mg/dl	0.9 ± 0.5	0.9 ± 0.4	1.0 ± 0.5	1.2 ± 1.0	1.0 ± 0.6
GFR, ml/min	139.5 ± 79.9	74.3 ± 32.4	75.1 ± 26.8	97.9 ± 42.1	95.8 ± 53.4
Austin activity score (0–24)	2.5 ± 0.9*	5.8 ± 1.5*	9.5 ± 4.2	1.7 ± 1.3*^†^	5.9 ± 4.5

The mean values are presented as mean ± SD

*p < 0.01 vs. ISN/RPS class IV; ^†^p < 0.05 vs. International Society of Nephrology/Renal Pathology Society (ISN/RPS) class III. *GFR* glomerular filtration rate, *GSI* glomerulosclerosis score, *TSI* tubular injury score

The use of formalin-fixed paraffin-embedded (FFPE) material from the archive of the Department of Nephropathology was approved by the Ethics Committee of the Friedrich-Alexander-University of Erlangen-Nürnberg, waiving the need for retrospective consent for the use of archived rest material (Re.-No. 4415).

More than half of the patients (56 %) received a treatment at the time of biopsy. Most of these treated patients received glucocorticoids (76 %) and 47 % received mycophenolate mofetil; 21 % of the treated patients received hydroxychloroquine or azathioprin. A few patients (<8 %) received cyclophosphamide, rituximab or methotrexate. In addition, most of the treated patients (85 %) received a combination of different medications.

### Immunohistochemical analysis and double-staining

In our study we used iNOS/CD68 as a marker for M1-like macrophages, which is well-established [19, 25–29]. Furthermore, we used CD206/CD68 to describe an M2a-like macrophage subtype and CD163/CD68 as a marker for an M2c-like subtype, as CD206 is highly expressed on the surface of M2a-like macrophages and CD163 is prominent on the surface of M2c-like macrophages (as described by Ohlson et al. [30]). For all co-localization studies kidneys were fixed in formalin, embedded in paraffin and cut into sections of 2 μm. Antigen retrieval was done using citrate buffer pH 6 and by cooking in a pressure cooker for 2.5 minutes. After blocking with normal goat serum and 1 % blotto, sections were incubated overnight at 4 °C using the following antibodies diluted in 1 % BSA in 50 mM Tris pH 7.4: iNOS, a rabbit polyclonal antibody against human iNOS (dilution 1:500) (ab15323; Abcam plc, Cambridge, UK); CD163, a mouse monoclonal antibody against human CD163 (dilution 1:500) (NCL-CD163; Novocastra, Leica Biosystems Newcastle Ltd; Newcastle, UK); CD206, a mouse monoclonal antibody against human CD206 (dilution 1:400) (H00004360; Abnova, Jhongli City, Taiwan); CD68, a mouse monoclonal antibody to human CD68 (dilution 1:50) (M0876; Dako Deutschland GmbH, Hamburg, Germany); and FoxP3, a monoclonal mouse antibody against FoxP3 (dilution 1:200) (ab236A; Abcam plc). Negative controls for immunostaining included either deletion or substitution of the primary antibody with equivalent concentrations of an irrelevant murine monoclonal antibody or pre-immune rabbit IgG. Double staining for CD68 and CD163, iNOS and CD206 was performed using the Alkaline phosphatase detection kit (POLAP-100, Zytomed Systems GmbH, Berlin, Germany) and Fast red (Sigma-Aldrich, Deisenhofen, Germany) as a substrate for CD163, iNOS and CD206 detection and Fast blue (Sigma-Aldrich) as a substrate for CD68 detection. For the FoxP3 double-staining with macrophage markers, the retrieval cooking time was extended to 5 minutes and Fast red was used to detect FoxP3 and Fast blue for macrophage markers.

### Quantitative and qualitative evaluation of macrophages and FoxP3+ cells in renal biopsies

CD68+/iNOS+, CD68+/CD206+ and CD68+/CD163+ double-positive, CD68+ cells, and FoxP3-positive cells were counted in the whole renal biopsy using a light-microscope at × 400 magnification. Stained sections were evaluated by counting cells per mm^2^ using computer assisted image analysis software (Count, Biomas, Erlangen, Germany) or by using a grid. Three different renal compartments were quantified separately: the glomerulus, the cortical tubulointerstitium and the medulla.

### Histopathological evaluation

Changes in glomerular morphology including mesangial matrix accumulation and sclerosis were evaluated by a semi-quantitative glomerulosclerosis score (GSI) and tubular interstitial changes were graded by the tubular injury score (TSI) using perjodate acid Schiff (PAS)-stained paraffin sections as described previously [31]. Interstitial fibrosis and tubular atrophy (IF/TA) was graded in line with the Banff classification for renal transplant biopsies [32]. Lupus disease activity and chronicity in lupus biopsies were graded by the Austin score [33]. In addition, the percentage of crescent formation was determined for each biopsy.

### Evaluation of retrospective clinical data

Clinical parameters from patients were retrospectively acquired from the time point of biopsy collection. The following parameters were included for correlation analysis of the evaluated macrophage subpopulations and injury scores using SPSS software: patient age, hypertension, diabetes, proteinuria, serum creatinine, serum urea, anti-nuclear antibody (ANA)-titer, anti-double-stranded DNA (anti-dsDNA) antibody titer, serum cholesterol, serum protein, and serum C3 and C4 complement.

### Statistical analyses

After testing for normal distribution of values using Kolmogorov-Smirnov test and finding that many datasets are not normally distributed, data were analyzed using the Kruskal-Wallis test and Dunn’s multiple comparison test post hoc test for comparison of SLE ISN/RPS classes. In all tests *p* < 0.05 was accepted as statistically significant. Most data are presented as scatter dot plots and only the ratios of M1-like to M2-like macrophages and Fig. 6 are presented as bars, all indicating the mean ± SEM. Spearman’s test was used to test correlation between renal macrophage infiltration and renal injury scores or clinical data. Association between macrophage infiltration and hypertension was tested using the Mann-Whitney rank test. Statistical analyses were performed using SPSS for Windows software (version 19.0 SPSS, IBM, Munich, Germany) or GraphPad Prism 5 for Windows software (version 5.02, GraphPad software Inc., San Diego, CA, USA).

Hierarchical cluster analysis (Euclidean distance, complete linkage) using data for macrophage counts per area in the glomeruli, cortex and medulla was performed in R software environment version 3.2.1 (R Core Team (2015) (R: a language and environment for statistical computing, R Foundation for Statistical Computing, Vienna, Austria (http://www.R-project.org/)). Prior to analysis three patients with missing values for macrophage counts were excluded and values of macrophage counts per area were standardized by calculating *Z* scores. Ten clusters of patients have been identified by cutting the resulting dendrogram.

## Results

### Characteristics of SLE patients with ISN/RPS class II–V

In this study biopsies from SLE patients with ISN/RPS class II-V were included (Table 1). Most of these patients were female (84 %). The proportion of male patients in the different SLE classes was not significantly different and the male-to-female ratio varied from 0.08 in class III to 0.31 in ISN/RPS class V (Table 1). In addition, the mean age of the SLE patients in this study at the time of biopsy was about 30 years in all four classes investigated (Table 1).

Hypertension was noted in 25–31 % of all patients. Diabetes was diagnosed only in a small number of patients with lupus who were allocated to ISN/RPS classes II and V. Renal function as assessed by serum creatinine, GFR and proteinuria varied widely within the ISN/RPS classes and therefore no significant differences were noted. There were no significant differences in any of the other criteria among patients in the different SLE classes investigated (Table 1). Evaluation of histopathological changes revealed significantly higher glomerulosclerosis in ISN/RPS class IV compared to all other investigated groups. However, glomerulosclerosis was comparable in ISN/RPS classes II, III and V (Table 1). In contrast, tubulointerstitial changes (TSI) (Table 1) were similar in all classes. Crescent formation was absent in ISN/RPS classes II and V and class IV biopsies had the highest prevalence of crescent formation (Table 1). The Austin activity index was highest in class IV followed by class III and was significantly lower in all classes compared to class IV (Table 1).

### Macrophage number and ratio of polarized macrophages is dependent on ISN/RPS class

**Fig. 1:**
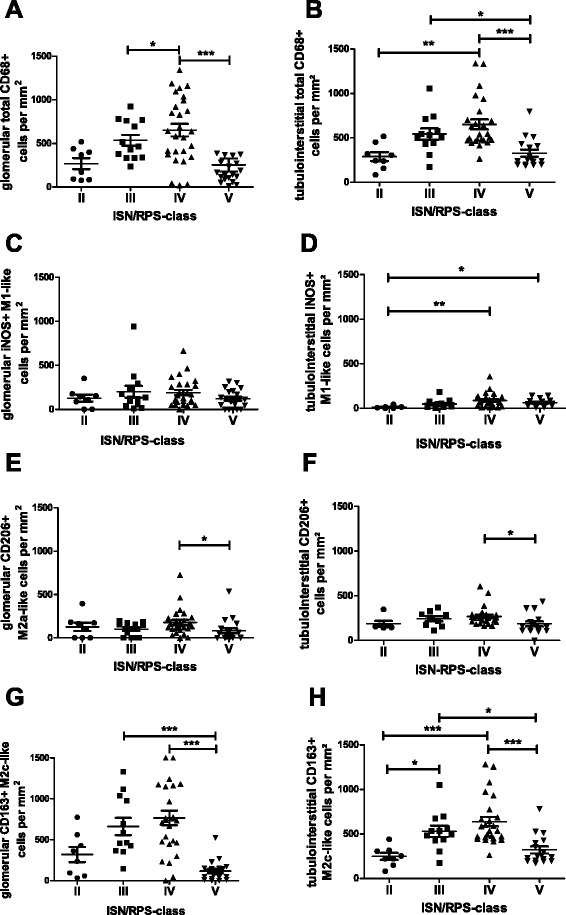
Distribution of macrophage subsets in different International Society of Nephrology/Renal Pathology Society (ISN/RPS) classes. Total CD68+, M1-like inducible nitric oxide synthase (iNOS)+/CD68+ double-positive, M2a-like CD206+/CD68+ double-positive and M2c-like double-positive CD163+/CD68+ macrophages were analyzed in renal biopsies from patient with lupus nephritis ISN/RPS classes II–V using immunohistochemical analysis. **a** Total glomerular CD68+ macrophages. **b** Total tubulointerstitial CD68+ macrophages. **c** Glomerular M1-like macrophages. **d** Tubulointerstitial M1-like-macrophages. **e** Glomerular M2a-macrophages. **f** Tubulointerstitial M2a-like macrophages. **g** Glomerulular M2c-like macrophages. **h** Tubulointerstitial M2c-like macrophages. Significant differences between ISN/RPS classes are marked with *lines* and *asterisks* (*p < 0.05; **p < 0.01; ***p < 0.001)

First, we analyzed total CD68+ macrophage infiltration in all four investigated SLE ISN/RPS classes. There was correlation between the number of infiltrating macrophages in the glomerular and tubulointerstitial compartments and SLE ISN/RPS class. The number of total CD68+ macrophages per glomerular area was greatest in class IV renal biopsies, with mean macrophage numbers about 2.5 times higher compared to SLE ISN/RPS class V (Fig. 1a; *p* < 0.001) and about 2.4 times higher compared to SLE ISN/RPS class II (Fig. 1a; *p* < 0.05). The fewest CD68+ glomerular macrophages were detected in biopsies from patients with SLE ISN/RPS class V, for which the mean was more than 2.0 times lower compared to values in patients with SLE ISN/RPS class III (Fig. 1a; *p* < 0.01). Differences in total macrophage infiltration in the tubulointerstitial compartment were less pronounced, with approximately two-fold maximal differences in mean values (Fig. 1b). However, there were significant differences when making the same comparisons of SLE ISN/RPS classes in relation to glomerular CD68 infiltration (Fig. 1b).

**Fig. 2:**
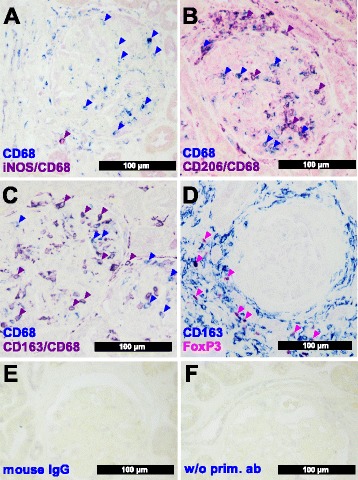
Immunohistochemical double-staining in different International Society of Nephrology/Renal Pathology Society (ISN/RPS) classes for macrophage subsets. Representative double-staining for the different macrophage subsets using immunohistochemical analysis are shown. **a** M1-like macrophages (example from ISN/RPS IV biopsy) were double-positive (*violet* and *violet arrowhead*) for inducible nitric oxide synthase (*iNOS*) (*red*) and CD68 (*blue* and *blue arrowheads*). **b** M2a-like macrophages (example from ISN/RPS IV biopsy) were double-positive (*violet*, e.g., *violet arrowheads*) CD206 (*red*) and CD68 (*blue* and *blue arrowheads*). **c** M2c-like macrophages (example from ISN/RPS III) were double-positive (*violet*, e.g., *viola arrowheads*) CD163 (*red*) and CD68 (*blue* and *blue arrowheads*). **d** Regulatory T cells (example from ISN/RPS IV biopsy) were positive for forkhead-box-protein P3 (*FoxP3*) (*purple* and *purple arrowheads*) and M2c-like CD163+ macrophages (*blue*). **e** Negative control using normal mouse IgG (example from ISN/RPS IV biopsy). **f** Negative control using secondary antibody only (example from ISN/RPS IV biopsy)

For characterization of macrophage polarization we performed immunohistochemical double staining combining the pan-macrophage marker CD68 with iNOS to detect M1-like macrophages (Fig. 2a), with CD206 to detect M2a-like macrophages (Fig. 2b) and with CD163 to detect M2c-like macrophages (Fig. 2c).

In all SLE ISN/RPS classes infiltration with iNOS-positive M1-like macrophages was comparable in the glomerular compartment but higher in number compared to the tubulointerstitial compartment (Fig. 1c). In the tubulointerstitial compartment M1-like macrophages were most prevalent in SLE ISN/RPS class IV biopsies, with significantly greater numbers compared to renal biopsies from patients with class II disease (Fig. 1d, *p* < 0.01). The smallest mean number of M1-like macrophages was observed in SLE ISN/RPS class II renal biopsies, and this was significantly smaller than the number of M1-like macrophages in class V renal biopsies (Fig. 1d; *p* < 0.05). There were also few M2a-like CD206+ macrophages in the glomeruli but slightly more in the tubulointerstitial compartment compared to M1-like macrophages (Fig. 1c-f). When comparing SLE ISN/RPS classes, significantly more M2a-like macrophages were found only in class IV vs. V (*p* < 0.05; Fig. 1f). The CD163+ M2c-like macrophage was the dominant subtype in all investigated SLE classes (Fig. 1). The numbers of M2c-like macrophages within the tubulointerstium were significantly different between the investigated ISN/RPS classes (Fig. 1h). However, some differences were not statistically significant due to greater variance within the glomerular compartment (Fig. 1g).

**Fig. 3:**
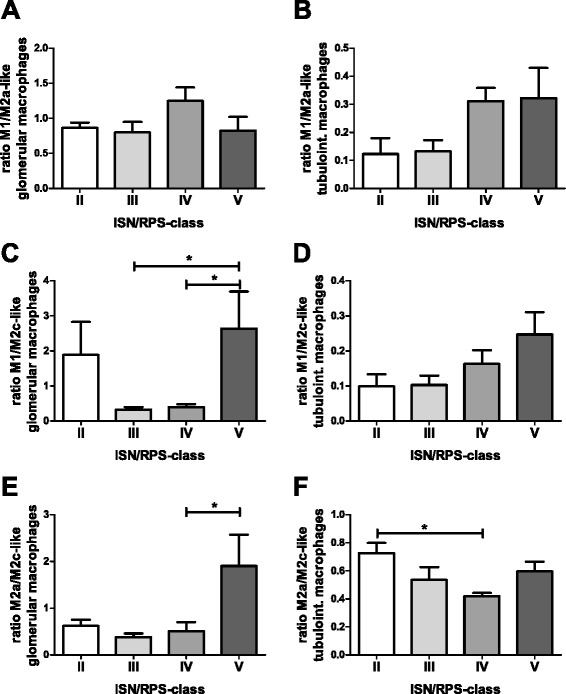
Ratios for the different macrophage subsets in each International Society of Nephrology/Renal Pathology Society (*ISN/RPS*) class. **a** Glomerular ratio for M1-/M2a-like macrophages. **b** Tubulointerstitial ratio for M1-/M2a-like macrophages. **c** Glomerular ratio for M1-/M2c-like macrophages. **d** Tubulointerstitial ratio for M1-/M2c-like macrophages. **e** Glomerular ratio for M2a-/M2c-like macrophages. **f** Tubulointerstitial ratio for M2a-/M2c-like macrophages. Significant differences are marked with *lines* and *asterisks* (*p < 0.05)

Interestingly, the ratios of M1-like to M2a-like and M2c-like macrophages, which are indicators of changes in the inflammatory milieu, were also dependent on SLE ISN/RPS class (Fig. 3). The differences in the ratios of M1/M2a-like macrophages were not significant (Fig. 3a), but in the tubulointerstitial compartment mean values of M1/M2a-like ratios had a strong tendency towards higher ratios that were more than doubled in class IV and V compared to ISN/RPS classes II and III (Fig. 3b). Glomerular ratios of M1/M2c-like macrophages were more than six times lower in SLE ISN/RPS class III and IV compared to ratios in class V renal biopsies (*p* < 0.05; Fig. 3c). However, the ratios of M1/M2c-like macrophages in the tubulointerstitium were not significantly different in the investigated SLE ISN/RPS classes (Fig. 3d). In addition, we also observed differences in M2a/M2c-like macrophage ratios. The glomerular M2a/M2c-like macrophage ratios in SLE ISN/RPS class V was significantly higher compared to ratios observed in class IV (*p* < 0.05; Fig. 3e). In contrast, in the tubulointerstitium the M2a/M2c-like macrophage ratio was significantly lower in SLE ISN/RPS class IV compared to class II, indicating compartment-specific differences in the ratios of polarized macrophages (*p* < 0.05; Fig. 3f).

### Clustering of macrophage subpopulations in different renal compartments in biopsies from patients with lupus nephritis

**Fig. 4:**
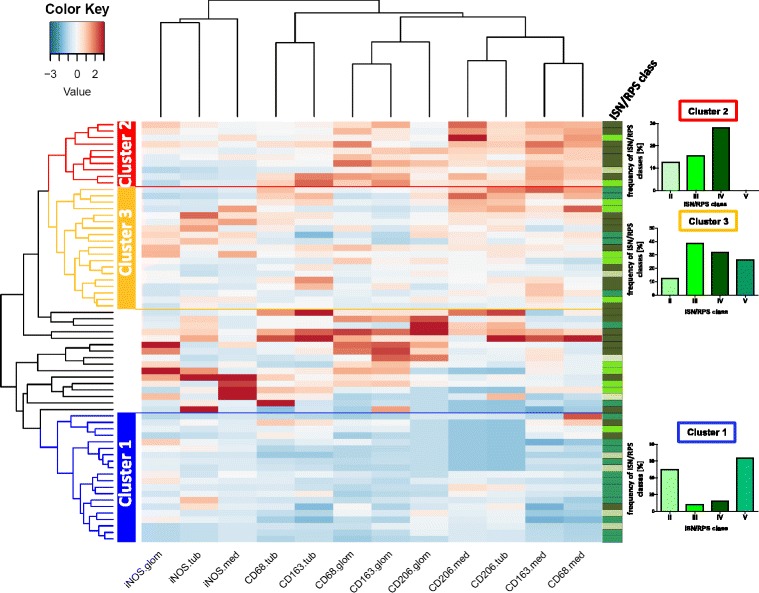
Clustering of patients with lupus nephritis using frequencies of different macrophage subsets. Patients with diagnosed lupus nephritis were clustered on the basis of the profiles of macrophage subsets in different renal compartments. International Society of Nephrology/Renal Pathology Society (*ISN/RPS*) systemic lupus erythematosus (SLE) classes of biopsies in each cluster are indicated using a color code (*pale green* class II, *light green* class III, *olive green* class IV, *viridian* class V). The frequencies of SLE ISN/RPS classes in the clusters are shown as the number of cases within the cluster per total number of patients allocated to this class in this study. *iNOS* inducible nitric oxide synthesase

Next we asked if the information on macrophage subtypes and its frequencies in different renal compartments could be used to confirm the classical histomorphological classification or is useful for the description of a new classification. To this aim patients were clustered on the basis of the macrophage subpopulation profiles (Fig. 4). In the heatmap in the figure each row corresponds to a patient, while the columns represent the abundance of different macrophage subtypes in the renal compartments (glomeruli, tubulointerstitium, and medulla), visualized by a color code ranging from blue (low) to red (high). Interestingly, three major groups with different macrophage subpopulation profiles were identified by cluster analysis. Each cluster included patients who could be allocated to more than one ISN/RPS class. However, two clusters (cluster 1 + 2) partially reflected the classical histomorphological classification. Cluster 1 contains more than 50 % of patient biopsies allocated to classes II and V. This cluster is characterized by low macrophage infiltration in all renal compartments. Cluster 2 is dominated by patients of lupus ISN/RPS class IV. It contains nearly 30 % of all ISN/RPS class IV patients and also some patients of ISN/RPS class II and III. This cluster is characterized by high infiltration of M2a and M2c-like macrophages in all renal compartments. In cluster 3 most biopsies are from patients with lupus nephritis class IV (n = 8), but the frequency was comparable to classes II and V, when calculated as the percentage of the respective ISN/RPS class cases included in this study. This cluster represents patients with more M1-like macrophages in the glomeruli, tubuli, and medulla compared to clusters 1 and 2 (Fig. 4).

### Loss of kidney function is associated with increased macrophage infiltration

There was positive correlation between loss of kidney function, as assessed by creatinine clearance, serum creatinine and serum urea, and the number of several tubulointerstitial macrophage subtypes, but not with glomerular macrophage infiltration. There was strongly significant correlation between serum creatinine and the number of tubulointerstitial total CD68+ (*r* = 0.411), CD206+ M2a-like macrophages (*r* = 0.441), and CD163+ M2c-like macrophages (*r* = 0.414) (Table 2). Furthermore, in this compartment there was positive correlation between creatinine clearance and the number of total CD68+ macrophages (*r* = 0.454) and M2c-like, CD163+ macrophages (*r* = 0.617) (Table 2). In addition, there was correlation between serum urea and total CD68+ (*r* = 0.621) and both M2a-like (*r* = 0.506) and M2c-like (*r* = 0.611) tubulointerstitial macrophages. In contrast, we found no correlation between proteinuria and the number of any macrophage subtype.

**Table 2:** Correlation between tubulointerstitial macrophage subpopulations and renal function

	Total	M1-like	M2a-like	M2c-like
	CD68+	iNOS+/CD68+	CD206+/CD68+	CD163+/CD68+
Creatinine clearance, ml/min (n = 20)	*r* = –0.454*	*r* = 0.064	*r* = –0.302	*r* = –0.617**
	*p* = 0.044	*p* = 0.788	*p* = 0.209	*p* = 0.007
Serum creatinine, mg/dl (n = 52)	r = 0.411**	*r* = -0.012	*r* = 0.441**	*r* = 0.414**
	*p* = 0.002	*p* = 0.931	*p* = 0.001	*p* = 0.002
Serum urea, mg/dl (n = 34)	*r* = 0.621**	*r* = 0.042	*r* = 0.506**	*r* = 0.611**
	*p* < 0.001	*p* = 0.809	*p* = 0.002	*p* < 0.001

Significant correlation *p < 0.05; **p < 0.01. *iNOS* inducible nitric oxide synthase

### Increased macrophage infiltration is associated with renal injury and fibrosis

**Fig. 5:**
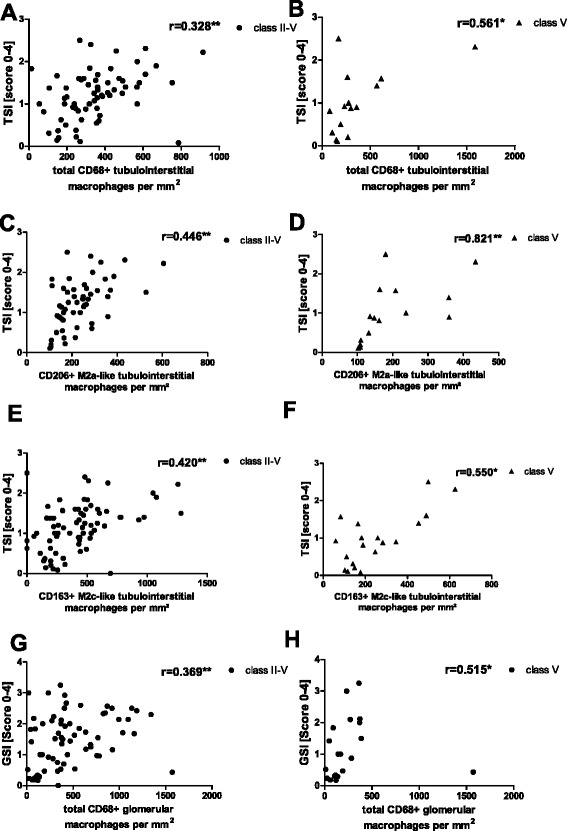
Tubular injury score (*TSI*) and glomerulosclerosis score (*GSI*) correlation with macrophage subtypes. TSI and GSI analyzed in renal biopsies from patients with lupus nephritis correlated with renal total, M2a-like and M2c-like macrophage invasion when analyzed by the Spearman test independently of the International Society of Nephrology/Renal Pathology Society class (**a**, **c**, **e**, **g**) and restricted to lupus nephritis (LN) class V (**b**, **d**, **f**, **h**). **a** Correlation between TSI in all patients with LN and total CD68-positive macrophages. **b** Correlation between TSI in patients with LN class V and total CD68-positive macrophages. **c** Correlation between TSI in all patients with LN and M2a-like macrophages. **d** Correlation between TSI in patients with LN class V and M2a-like macrophages. **e** Correlation between TSI in all patients with LN and M2c-like macrophages. **f** Correlation between TSI in patients withg LN class V and M2c-like macrophages. **g** Correlation between GSI in all patients with LN and total CD68-positive macrophages. **h** Correlation between GSI and patients with LN class V and total CD68-positive macrophages. Significant correlation is marked by asterisks (*p < 0.05; **p < 0.01)

In our cohort of patients with SLE we found that renal injury is associated with most of the investigated macrophage subtypes. In the tubulointerstitium there was positive correlation between a higher TSI and the number of total CD68+ macrophages (*r* = 0.328; Fig. 5a) but also with the number of M2a-like (*r* = 0.446; Fig. 5c) and M2c-like macrophages (*r* = 0.420; Fig. 5e) when correlation with the TSI was tested independently of ISN/RPS lupus class. In contrast, there was no correlation between the TSI and the number of M1-like macrophages. Interestingly, there was stronger correlation with TSI when the analysis was restricted to class V patients (Fig. 5b, d, f). Sub-analysis of other lupus nephritis (LN) classes revealed only significant correlation) between TSI and M2c-like macrophages in ISN/RPS class IV patients (*r* = 0.550, *p* = 0.015). Furthermore, a higher GSI correlated with the number of most of the investigated macrophage subtypes within the glomeruli, including total CD68+ macrophages (*r* = 0.369; Fig. 5g), CD206+ M2a-like macrophages (*r* = 0.280; *p* < 0.05), CD163+ M2c-macrophages (*r* = 0,333; *p* < 0.01) and only weakly with iNOS+ M1-like macrophages (*r* = 0.263; *p* = 0.03). Again, on sub-analysis of LN class V patients, there was stronger correlation between total CD68+ macrophages and GSI (Fig. 5h). There was weak correlation between crescent formation (a sign of active glomerular damage) and the number of glomerular total CD68+ macrophages (*r* = 0.302; *p* = 0.012) and between crescent formation and glomerular CD163+ M2c-like macrophages (*r* = 0.318, *p* = 0.008). Interestingly, there was strong positive correlation between the Austin activity score (Table 1) and glomerular (*r* = 0.549; *p* < 0.001) and tubulointerstitial (*r* = 0.550; *p* < 0.001) CD68+ macrophages and M2c-like glomerular (*r* = 0.382; *p* = 0.005) and tubulointerstitial macrophages (*r* = 0.525; *p* < 0.001) but no correlation between Austin score and M1-like and M2a-like macrophages in either compartment. Macrophages are also known to exert pro-fibrotic properties; therefore, we investigated interstitial fibrosis and tubular atrophy (IFTA) in renal biopsies. The number of tubulointerstitial M2a-like and M2c-like macrophages were similarly correlated (*r* = 0,360 and 0,355, *p* < 0,005) with IFTA.

### Correlation of anti-dsDNA antibody titer and complement C3 and C4 consumption with the number of different macrophage subtypes

Interestingly, there was positive correlation between SLE-related anti-dsDNA antibody titers and different macrophage subpopulations in the glomerular and tubulointerstitial compartment. There was correlation between the anti-dsDNA antibody titers and the number of total CD68+ macrophages only in the glomerular (*r* = 0.369; *p* = 0.035, n = 31) and not in the tubulointerstitial compartment. In contrast, correlation between anti-dsDNA antibody titer and iNOS+ M1 macrophages (*r* = 0.412; *p* = 0.019, n = 32) was observed only in the tubulointerstitium. There was comparable correlation between anti-dsDNA antibody titers and the numbers of glomerular CD163+ M2c-like macrophages (*r* = 0.408; *p* = 0.018, n = 31) and both glomerular (*r* = 0.374; *p* = 0.032, n = 31) and tubulointerstitial CD206+ M2a-like macrophages (*r* = 0.467; *p* = 0.008, n = 31). In patients with lupus nephritis the plasma levels for complement C3 and C4 are decreased due to renal complement deposition. Here, we observed a negative correlation between plasma complement C3 and the number of total glomerular CD68+ macrophages (r = –0.412; *p* = 0.004, *n* = 45) and stronger correlation between complement C3 glomerular CD163+ M2c-like macrophages (*r* = –0.556; *p* < 0.001, n = 45). Interestingly, there was negative correlation between the number of CD163+ M2c-like macrophages and plasma C4 levels (*r* = –0.367; *p* = 0.022, *n* = 43). In the tubulointerstitium we observed no correlation between complement C3 or C4 plasma levels and any macrophage subtype.

### Increased M2a-like macrophage infiltration is associated with hypertension

**Fig. 6:**
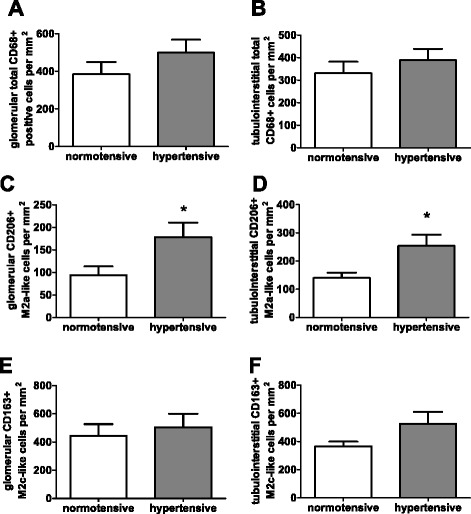
Association between hypertension and macrophage subtypes. Numbers of total CD68-positive, M2a-like and M2c-like macrophages were analyzed in normotensive and hypertensive patients with lupus nephritis. **a** Comparison of glomerular total CD68-positive macrophage infiltration. **b** Comparison of tubulointerstitial total CD68-positive macrophage infiltration. **c** Comparison of glomerular M2a-like macrophage infiltration. **d** Comparison of tubulointerstitial M2a-like macrophage infiltration. **e** Comparison of glomerular M2c-like macrophage infiltration. **f** Comparison of tubulointerstitial M2c-like macrophage infiltration. Significant differences, as assessed by the Mann-Whitney *U* rank test, are marked by *asterisks* (*p < 0.013)

In biopsies from patients with SLE and clinically diagnosed hypertension, only the number of total CD206+ M2a-like macrophages in glomeruli and the tubulointerstitium was significantly higher than in normotensive patients with SLE (Fig. 6c, d). This association was restricted to M2a-like macrophages. There was no association between hypertension and glomerular and tubulointerstitial total CD68+ macrophages (Fig. 6a, b), M2c-like macrophages (Fig. 6e, f) or all M1-like macrophages (data not shown).

### FoxP3+ T regulatory cells co-localize with different macrophage subtypes

**Fig. 7:**
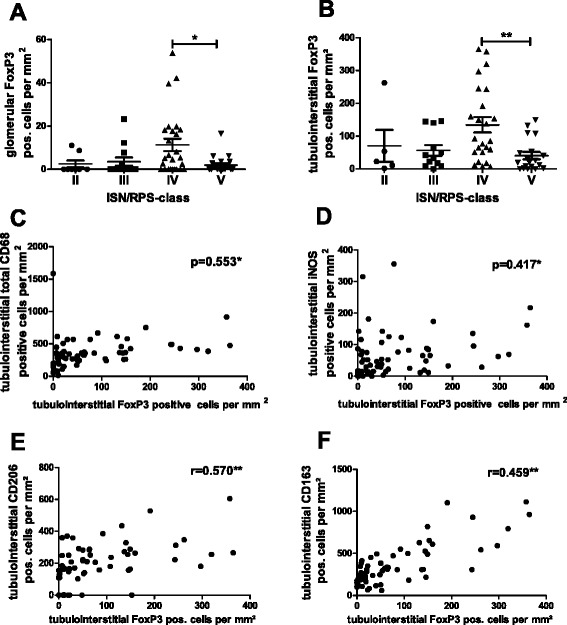
Distribution of T regulatory cells and their correlation with macrophage subtypes. The number of forkhead box protein P3 (*FoxP3*) + regulatory T cells was analyzed in International Society of Nephrology/Renal Pathology Society (*ISN/RPS*) lupus classes and correlation tested with numbers of total CD68+, M1-like, M2a-like and M2c-like macrophages in the tubulointerstitium. **a** Glomerular FoxP3+ cells in ISN/RPS class II–V. **b** Tubulointerstitial FoxP3+ cells in ISN/RPS classs II–V. **c** Correlation between FoxP3+ cells and tubulointerstitial total CD68+ macrophages. **d** Correlation between FoxP3+ cells and tubulointerstitial M1-like macrophages. **e** Correlation between FoxP3+ cells and tubulointerstitial M2a-like macrophages. **f** Correlation between FoxP3+ cells and tubulointerstitial M2c-like macrophages. Significant differences and correlation are marked by *asterisks* (*p < 0.05; **p < 0.01)

FoxP3+ regulatory T cells were detected in low numbers in glomeruli (Fig. 7a). More FoxP3+ cells were found in the tubulointerstitium (Fig. 7b). In both compartments the greatest number of FoxP3+ cells was found in SLE ISN/RPS class IV biopsies (Fig. 7a, b). FoxP3+ cells always co-located with macrophages, e.g., CD163+ M2c-like macrophages (Fig. 2d). However, the number of FoxP3+ T regulatory cells was positively correlated with all investigated macrophage subpopulations, M1-like (Fig. 7d), M2a-like (Fig. 7e), M2c-like (Fig. 7f) and consequently the number of total CD68+ macrophages in the tubulointerstitial compartment (Fig. 7c).

## Discussion

### Macrophage subpopulations differ in lupus nephritis ISN/RPS classes

In this study we investigated macrophage subpopulations in different ISN/RPS classes of lupus nephritis for the first time. The first major finding in our study is that infiltration of macrophage subpopulations differs between the four investigated lupus nephritis ISN/RPS classes and is dominated by CD163+ M2c-like macrophages. Interestingly, macrophage infiltration differs particularly in the number of M2c-like macrophages; differences among the investigated ISN/RPS classes in M1-like and M2a-like macrophage infiltration were only minor. However, the proportion of M1-like macrophages to M2c-like macrophages was greater in the glomeruli but not in the tubulointerstitium of ISN/RPS classes II and V (Fig. 4, cluster 3) indicating compartment-specific differences in macrophage subtype composition. Both the significant differences in number of macrophage subtypes and the cluster analysis using the frequencies of macrophage subtypes in different renal compartments support the thesis that the classical ISN/RPS classes for lupus nephritis can be characterized by a specific macrophage signature, as single classes dominate the clusters. In addition, the cluster analysis allows further refinement in the characterization of a lupus class. For example, ISN/RPS class IV biopsies can be divided into a pro-inflammatory subclass with greater involvement of M1-like macrophages found in cluster 3 and an anti-inflammatory M2-like dominated subclass collected in cluster 2. We suggest that cluster 3 may reflect the macrophage infiltration during initiation of lupus nephritis and chronic alterations in the kidney were found in cluster 2. However, we did not find any differences in disease activity when these data were compared with the Austin activity score.

### Why is the M2c-like subtype the dominant macrophage subtype in human lupus nephritis?

The second major finding is that the predominant macrophage subtypes are M2-like macrophages. The dominant macrophage subtype in biopsies from patients with SLE was the M2c-like subtype, designated as deactivated, remodeling or anti-inflammatory macrophages. High numbers of CD163+ macrophages in class IV lupus nephritis were also recently observed by Li et al. [34]. The M1-like macrophage subtype has long been believed to be a source of pathology in lupus nephritis [25] and the high levels of IL-6 [35, 36], IFN-ɣ [35] and TNF-α [36] found in patients with lupus nephritis support the important role of pro-inflammatory M1-like macrophages in the pathogenesis of the disease. There are two possible explanations that could account for the relatively low numbers of M1-like macrophages. First, sampling of human biopsies in early disease is probably much rarer than in murine biopsies. Therefore, there may be fewer M1-like macrophages at the first indication for biopsy in patients compared to mouse models in which the timinge of sampling is dependent on the experimental setting and not on the clinical need for diagnosis. Second, the small number of pro-inflammatory M1-like macrophages is probably a consequence of the immunosuppressive therapy, as nearly all substances that are used for therapy in our study are known to increase CD163 expression and thus an M2 phenotype. Glucocorticoids, the most frequently used therapy in our study, have been shown to increase CD163 on fetal macrophages [37], in a lung transplantation model [38], and in kidney allograft transplantation [39]. Guo et al. describe a conversion of macrophages into an M2 phenotype driven by an immunosuppressive regimen using mycophenolate mofetil [40]. In addition, rituximab and cyclophosphamide therapy were also shown to upregulate CD163 on macrophages [41]. The M2c-like subtype is further driven by IL-10 [17], a cytokine that is highly upregulated in patients with lupus nephritis [36, 42], suggesting this macrophage subtype is highly relevant in this disease. On the other hand, clearing of apoptotic cells, a frequently observed phenomenon in lupus nephritis, requires differentiation of macrophages to an M2c-like phenotype [43]. Furthermore, high concentrations of soluble CD163 released by M2c-like macrophages can be found in plasma from patients with SLE and correlate with the SLE disease activity index [22], indicating that this macrophage subtype is highly induced in active disease. This is in accordance with our data, establishing the CD163+ macrophages as the dominant renal macrophage subtype in human lupus nephritis.

### Is infiltration with specific macrophage subpopulations associated with or causative of renal disease?

The correlation between infiltrating macrophage subpopulations and renal function, injury and lupus-nephritis-specific pathology was the third major finding. The relevance of macrophages for the pathogenesis of lupus nephritis has been shown in several studies [12–16], e.g., there is correlation between the numbers of infiltrating CD68+ macrophages and monocytes and clinical outcome parameters, such as proteinuria, in human lupus nephritis [44]. However, the relevance of specific macrophage subpopulations remained unclear. Interestingly, in our study a decrease in renal function was associated with an increase in M2-like macrophages but not in M1-like macrophages. There was correlation between both M2a-like and M2c-like macrophages and nearly all markers of renal functional impairment, despite a much lower prevalence of renal M2a-like macrophages. Loss of kidney function is associated with increasing kidney fibrosis. Here, we confirmed correlation between the numbers of M2a-like and M2c-like macrophages and TSI, GSI, crescent formation, and IFTA, indicating that both cells types might be involved in fibrinogenesis. Interestingly, correlation of M2 subtype macrophage numbers with renal scarring was strongest in patients in ISN/RPS class V. This class is characterized by the highest proteinuria levels and reduced kidney function (Table 1) but relatively low total macrophage numbers compared to class IV, indicating that M2-like macrophages are more relevant for disease modulation or progression in this low-inflammatory state of lupus nephritis class V. There was also positive correlation between the number of M1-like macrophages and the GSI, but on a lower level and with lower significance. Although M2-like macrophages are known to be immunomodulatory or anti-inflammatory, there are several studies confirming a role of M2-like macrophages in disease progression. Our observation that CD163+ macrophage infiltration was associated with decreased renal function was confirmed for other renal disease like IgA-nephropathy [45] and in patients after kidney transplantation [46]. However, it remains unclear whether CD163+ cells are actors of disease progression, or recruited to counteract this process in order to prevent progressive inflammation. There are some hints that CD163+ macrophages are actively involved in disease progression, as suggested in a study investigating CD163 in skin lesions from patients with SLE [47]. In addition, both M2a-like [48] and M2c-like subtypes [49] express transforming growth factor (TGF)-ß, a well-known pro-fibrotic cytokine which might well argue for a leading role of both macrophage subtypes in scarring, as supported by the correlation of M2-like macrophages with IFTA as observed here.

### M2a-like macrophage infiltration is associated with hypertension

Hypertension is a major risk factor for the progression of renal and cardiovascular diseases and is the primary cause of mortality in SLE [50]. The exclusive association of M2a-like macrophages with hypertension as a marker of disease progression is the fourth major finding. As a result of chronic kidney disease (CKD) patients frequently develop hypertension. Despite little being known about the pathogenesis of hypertension in SLE, there are many studies supporting the involvement of the immune system [51]. Going in line with this notion, development of hypertension was prevented in different models by blocking of macrophage activation using a CCR2 antagonist [52] or macrophage depletion [53]. In a recent study, the IL-6/IL-31-signaling axis was found to be critical for the pathogenesis of pulmonary arterial hypertension, with evidence of a strong association with M2-like macrophage infiltration [54]. Furthermore, vascular remodeling induced by angiotensin II was found to be mediated by M2-like macrophage polarization via class A scavenger receptor A activation [55]. Therefore, it appears possible that the higher numbers of M2a-like macrophages in hypertensive compared to normotensive patients with SLE indicates functional involvement of this macrophage subtype in the pathogenesis of hypertension. This hypothesis, however, needs further investigation and validation.

### Association between FoxP3+ cells and macrophage subtypes

FoxP3+ regulatory T cells (Treg) were shown to suppress inflammatory response by polarizing macrophages towards an M2c-like phenotype via IL-10 [56]. Therefore, we compared the numbers of macrophage subtypes and of FoxP3+ Tregs. Surprisingly, there was no exclusive correlation between FoxP3+ Treg and M2c-like macrophages in renal biopsies from patients with lupus nephritis, but simply an association with higher numbers of macrophages in general. Therefore, FoxP3+ cells appear to play a minor role in the induction of M2c-like macrophages. The use of FoxP3 as a single marker of Treg may not suffice to characterize Treg, as different Treg subpopulations have recently been described, and an imbalance of different types of CD4+ FoxP3+ cells was suggested to be involved in worsening kidney function in renal transplant rejection [57].

### Limitations of the study

Our study has some limitations. Investigating renal biopsies from human samples can only represent a snapshot of the current disease state. Therefore, we have no information on the dynamics of macrophage subtypes in early disease and during disease progression. The classification of macrophage subtypes used in this study is very simple, and may need to be refined in future studies. Animal studies using three different models for lupus nephritis identified two different macrophage subtypes and three different dendritic cell subtypes in diseased mice, but the dominant macrophage subtype could not be allocated to M1-like or M2-like macrophages [24]. When we observe the association between macrophage subtypes and, e.g., kidney injury, kidney function, or hypertension, it remains unclear whether macrophages are the cause or the consequence of these pathological alterations. Therefore, future experimental studies are needed to address and hopefully clarify these questions.

## Conclusions

In conclusion, our data on macrophages in different ISN/RPS classes of lupus nephritis show that these classes can be characterized by different macrophage subpopulations in the renal biopsy. The M2c-like macrophage is the dominant macrophage subpopulation in the tubulointerstitial compartment of all investigated ISN/RPS lupus classes. However, in the glomerular compartment the ratios of M1-like/M2c-like macrophages differ between classes, with more M1-like/M2c-like macrophages in classes II and V. Renal injury and function frequently correlated with numbers of M2a-like as well as M2c-like macrophages, while correlations with the number of M1-like macrophages were rarely found. The M2a-like macrophage subpopulation was exclusively associated with hypertension in our cohort of lupus nephritis patients. Hereby, we suggest that especially M2-like macrophage subpopulations may be important mediators of lupus nephritis.

## Abbreviations

BSA: bovine serum albumin
CCR2: chemokine receptor type 2
ERK: extracellular signal related
FFPE: formalin-fixed paraffin-embedded
FoxP3: forkhead-box-protein P3
GFR: glomerular filtration rate
GSI: glomerulosclerosis score
IF: interstitial fibrosis
IFTA: International Fibrosis and Tubular Atrophy
IL: interleukin
INF-ɣ: interferon gamma
iNOS: inducible nitric oxide synthase
ISN/RPS: International Society of Nephrology/Renal Pathology Society
LN: lupus nephritis
LPS: lipopolysaccharide
SLE: systemic lupus erythematosus
TA: tubular atrophy
TNF-α: tumor necrosis factor alpha
Treg: regulatory T cells
TSI: tubular injury score
